# Discrete Element Modelling of the Mechanical Behavior of Sand–Rubber Mixtures under True Triaxial Tests

**DOI:** 10.3390/ma13245716

**Published:** 2020-12-15

**Authors:** Yiming Liu, Xinchao Liao, Lihua Li, Haijun Mao

**Affiliations:** 1 School of Civil Engineering, Architecture and Environment, Hubei University of Technology, Wuhan 430068, China; ymliu@hbut.edu.cn (Y.L.); xcliao@hbut.edu.cn (X.L.); 2 Institute of Rock and Soil Mechanics, Chinese Academy of Sciences, Wuhan 430071, China; hjmao@whrsm.ac.cn; 3 The State Key Laboratory of Geomechanics and Geotechnical Engineering, Wuhan 430071, China

**Keywords:** discrete element method, intermediate principle stress, force transmission, fabric anisotropy, sand–rubber mixtures

## Abstract

Sand–rubber mixtures (SRMs) consisting of stiff sand particles and soft rubber particles are typical binary mixture materials that possess a variety of complicated properties. The complexity of the properties of sand–rubber mixtures is increased when complex stress path is involved. This study investigates the mechanical behavior of sand–rubber mixtures under generalized loading conditions using the discrete element method. A series of numerical true triaxial shear tests were conducted on pure sand and sand–rubber mixtures. The effect of rubber content and loading path on both of the macroscopic and microscopic performances of sand–rubber mixtures was investigated, and the associated microscale mechanism was also discussed. Numerical simulations show that the relationship between the peak friction angle $\phi_{p}$ and the intermediate principal stress ratio b is influenced by the addition of rubber particles, and a suggested explanation of this phenomenon is that the rubber particles mainly affect the inherent stability of the strong network. Particle-scale observations, including the coordinate number, the proportion of strong contacts, and the fabric anisotropy, are also presented in this study. Microscopic results confirm the explanation above, and explore the force transmission characteristics of sand–rubber mixtures under generalized loading conditions. This research can provide a reference for the constitutive model development of sand–rubber mixtures.

## 1. Introduction

Sand–rubber mixtures (SRM) recently have received considerable amount of interest from both the research community and practicing engineers, and have been widely used in geotechnical engineering, including lightweight backfill, retaining walls, highway embankments, and road construction, due to its light weight, high damping, and high permeability [1,2,3,4,5,6]. Sand–rubber mixtures are unconventional geo-material and possess a variety of unique properties. The mechanical behavior of sand–rubber mixtures depends not only on the properties of host sands but also on the characteristics of rubber particles. A full understanding of the mechanical properties of sand–rubber mixtures can provide a solid backup for the application of sand–rubber mixtures in geotechnical projects.

A lot of experimental and numerical research has been performed to investigate the mechanical behavior of sand–rubber mixtures. Previous results show that the mechanical behavior of sand–rubber mixtures is significantly influenced by the rubber content, by the size ratios of rubber to sand particles, and by the shapes of sand and rubber particles. For example, it is reported that the peak strength of sand–rubber mixtures increases when tyre shreds are used [7,8,9,10,11], while rubber crumbs either have no effect on or decrease the peak strength of sand–rubber mixtures [12,13,14]. The shear stiffness and critical state performance of sand–rubber mixtures can also be influenced by rubber sizes. Both the laboratory data and DEM simulation results show that $G_{max}$ decreases while rubber contents increase [15,16]. Lee et al. [17] investigates the mechanical properties of sand–rubber mixtures in the critical state and finds that the angle of shear resistance in the critical state deceases with an increase of rubber content. Fu et al. [8] reports that an increase of rubber content leads to a downward shift of the location of the critical state line in the volume–log mean stress plane. Fu et al. [11] investigates the critical state behavior of rubber–sand mixture materials with two different types of host sand. Lopera Perez et al. [18] comprehensively investigates the rubber size ratio on the critical state line and associated microscopic interpretation. Li et al. [14] experimentally examines the micro and macro-scale behaviors of sand–rubber mixtures with different types of host sand, and finds that adding rubber particles decreases the peak friction angle and increases the friction angle in the critical state.

Previous research has mainly focused on the mechanical behavior of the sand–rubber mixtures under conventional triaxial compression conditions, but the physical properties of sand–rubber mixtures under true triaxial shearing tests have seldom been reported. The effect of intermediate principal stress on the mechanical behavior of granular materials has been studied through laboratory tests and through numerical simulations [19,20,21,22,23,24,25,26,27,28,29]. The reported results show that the intermediate principal stress can significantly influence the mechanical behavior of granular materials, including shear strength, non-coaxiality, dilatant behavior, and shear bands.

Discrete element method (DEM), which was first developed by Cundall and Strack [30], has been proven to be a powerful tool in investigating the mechanical behavior of granular materials, and has been successfully applied to investigating the mechanical properties of sand–rubber mixtures [6,16,18,31] and to simulate the true triaxial shear tests [25,26,28]. This paper employed a three-dimensional discrete element simulation of sand–rubber mixtures with various rubber contents under true triaxial shear conditions. The effect of rubber particle on the shear strength and failure pattern of sand–rubber mixtures was investigated. The associated microscale mechanism was also discussed and analyzed. The findings in this study can provide a reference for the constitutive model development of sand–rubber mixtures.

## 2. Modelling Using the Discrete Element Method

### 2.1. Rolling Resistance Contact Model

The numerical simulations performed in this study use a commercial discrete element method (DEM) software named Particle Flow Code 3D (PFC3D) version 5.0, which is developed by the Itasca Consulting Group, Inc. [32]. PFC3D is one of the most famous DEM codes in the world, and has been widely used to investigate the macroscopic and microscopic responses of different kinds of geo-materials. In PFC3D, particles are treated as idealized spheres, and contact each other at contact points. This assumption ignores the rolling resistance aroused by particle shapes or other factors, and may lead to significant rotational inertia and energy loss [33,34,35]. To overcome this drawback, different rolling resistance models are proposed by adding a rotational frictional torque at the contact point to resist particle rotation [36,37,38]. In this study, the built-in rolling resistance linear model is applied to representing the particle contact behavior, while the linear contact model is used to model the particle-wall interaction.

The linear rolling resistance contact model is similar to the linear contact model, except that an internal moment is added to resist the relative rotation of the contact particles at the contact point. Normal and shear forces acting at contact points are calculated as:

(1) $$\{\begin{array}{c} F^{n}\;=\;k^{n}g_{s}\\ F^{s}\;=\;{\left(F^{s}\right)}_{0}\;-\;k_{s}\Delta \delta_{s} \end{array}$$

where $k^{n}$ and $k_{s}$ are the normal and shear stiffness, respectively, $g_{s}$ is the overlap, ${\left(F^{s}\right)}_{0}$ is the linear shear force at the beginning of the timestep, and $\Delta \delta_{s}$ is the adjusted relative shear-displacement increment which is nonzero only when $g_{s}$ is negative. The shear force follows a slip model, which is defined by the friction coefficient μ at the contact. The contact is sliding when $\left|F^{s}\right|\geq \mu \cdot F^{n}$ is satisfied. The rolling resistance moment $M^{r}$ is added to resist the relative rotation between particles, and it is calculated in the incremental form as follows:

(2) $$M^{r}=\;M^{r}-k_{r}\Delta \theta_{b}$$

where $\Delta \theta_{b}$ is the relative bend-rotation increment, and $k_{r}$ is the rolling resistance stiffness. There are many different definitions of $k_{r}$, and in this study $k_{r}$ is defined as:

(3) $$k_{r}=k_{s}{\bar{R}}^{2}$$

and $\bar{R}$ is the contact effective radius, which is defined as:

(4) $$\bar{R}\;=\;\frac{R^{\left(1\right)}\;R^{\left(2\right)}}{R^{\left(1\right)}\;+\;R^{\left(2\right)}}$$

where $R^{\left(1\right)}$ and $R^{\left(2\right)}$ are the radii of the contact ends. If one of the ends is a wall, then $R^{\left(2\right)}$ is infinite.

Similar to the sliding model, a rolling model is applied when the magnitude of rolling resistance moment exceeds a threshold limit, which is calculated as:

(5) $$\|M^{r}\|\;>\;M^{*}\;=\;\mu^{r}\;\bar{R}F^{n}$$

where $\mu^{r}$ is the rolling friction coefficient.

### 2.2. Model Generation and Loading Paths

**Figure 1:**
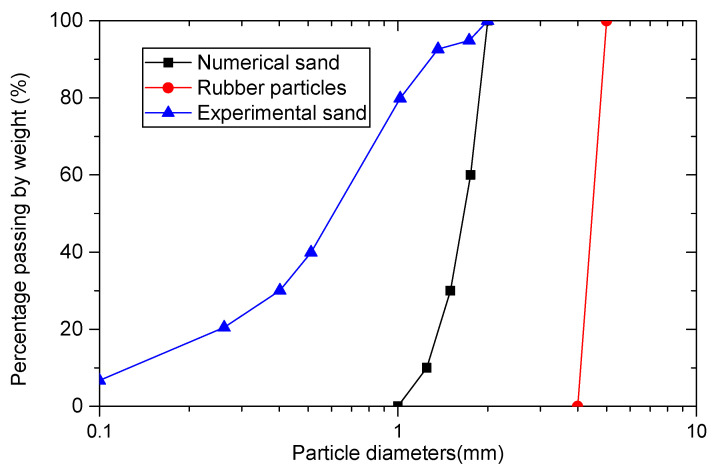
Particle size distribution of sand–rubber mixtures and numerical model.

To simulate the behavior of sand–rubber mixtures in generalized stress paths, a numerical true triaxial shear test sample is established. The numerical sample is enclosed by six rigid, frictionless walls with a size of 80 mm × 39.1 mm × 39.1 mm. The top and bottom walls are used as loading platens. The particle size distribution (PSD) of the simulated sand–rubber mixtures is similar to that of the materials reported by Deng et al. [39]. Deng et al. [39] and Gong et al. [31] can be referred to for a brief description of the sand–rubber mixtures and the background of the laboratory testing. The particle size distribution curves of sand and rubber are shown in Figure 1. The PSD of the simulated rubber matches that of Deng et al. [39] following a uniform distribution with minimum and maximum sizes of 4 mm and 5 mm. For numerical assemblies containing small, stiff particles, the computation times could be extremely long if the actual sizes were used. To increase the computation efficiency and reduce the calculation time, model scaling is necessary. In general, there are three primary methods of model scaling [40]: density scaling [41,42], gravity scaling [43,44], and mass scaling [45,46]. In the density scaling method, particle density increases by several orders of magnitude, while the particle sizes remain the same. The mass scaling method is just the opposite of the density scaling method. In the mass scaling method, particle sizes increases, while the particle density remain constant. The gravity scaling method increases gravity field, and keep the particle size and particle density constant. The mass scaling method has been proved to be suitable for sand–rubber mixtures [47], and has been successfully introduced into the DEM simulation of sand–rubber mixtures under different loading conditions [47,48]. In the current research, the mass scaling [40] was applied to scaling the numerical sand particles for the sake of the computation efficiency.

There are two phases of the preparation of the particle assembly. In the first phase, the sand particles and rubber particles are initially generated with random locations in the box enclosed by six walls following the size distributions shown in Figure 1. Note that there may be large particle–particle overlaps in the particle assembly. To reduce the overlaps to an acceptable level, a very low value of inter-particle friction coefficient is set to the particles at first. The numerical particle assembly is solved to allow the particles to rearrange until an equilibrium and isotropic state is achieved.

**Figure 2:**
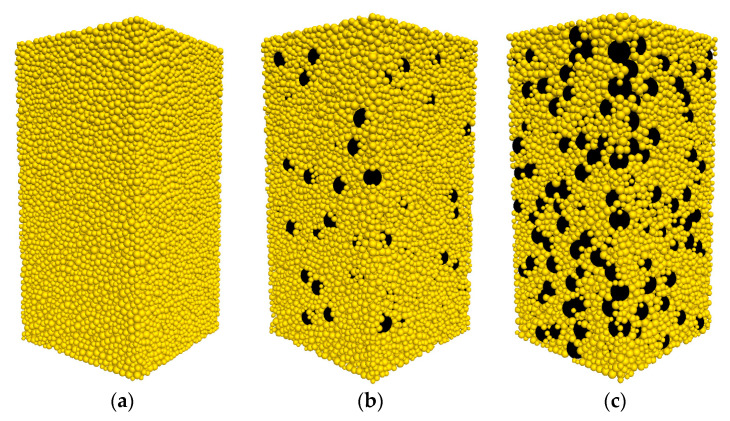
Numerical specimens of (**a**) pure sand, sand–rubber mixtures (**b**) with 10% rubber particles and (**c**) with 30% rubber particles. The yellow particles represent sand particles, and the black particles represent rubber particles.

In the second phase, the isotropically compressed sample is prepared by compressing the generated sample with a stress servo-control algorithm. The servo-controlled algorithm in PFC3D is called in each computational cycle to determine the current wall stresses and to adjust the wall velocities so as to reduce the difference between the current stress and the required stress. Sand particles are assigned an inter-particle friction coefficient (μ) of 0.01 while rubber particles are assigned a μ of 1.5 during the isotropic compression stage. The numerical samples with different rubber contents at the end of an isotropic compression of 100 kPa are shown in Figure 2.

Table 1 summarizes the simulations carried out in this study. Samples are labelled as CS-XXX-b for Clean Sand, with XXX indicating the confining pressure $\sigma_{3}$ and with b indicating the intermediate principle stress ratio. Samples are labelled as RS-RC-XXX-b for sand–rubber mixtures, with RC indicating the content of rubber in percentage, and similarly, with XXX indicating the confining pressure $\sigma_{3}$ and with b indicating the intermediate principle stress ratio. The numerical tests are conducted on dense sand–rubber mixtures ($D_{r}=70\%$) with different RC of 0%, 10%, and 30%. RC is the percentage of rubber particles by weight and is defined as $\mathrm{RC}=(m_{rubber}/m_{total})\times 100\%$, where $m_{rubber}$ is the mass of rubber particles and $m_{total}$ is the total mass of sand–rubber sample. The number of particles for specimens with different rubber content is also listed in Table 1.

Once the isotropic compression state is achieved, the numerical specimens are sheared under true triaxial shear conditions. In the true triaxial stress state, the major principal stress $\sigma_{1}$, the intermediate principal stress $\sigma_{2}$, and the minor principal stress $\sigma_{3}$ can be respectively manipulated to simulate the complex loading paths.

In this paper, the true triaxial shear tests under the constant $\sigma_{3}$ and constant intermediate principal stress ratio b westress state are presented. For the samples under the constant $\sigma_{3}$ and constant b stress state, $\sigma_{1}\;$ is coincident with the moving directions of the top and the bottom walls, and the value of $\sigma_{1}\;$ varies during the test. The intermediate principal stress $\sigma_{2}\;$ can be estimated from:

(6) $$\sigma_{2}\;=\;b\;\sigma_{1}\;+\;\left(1-b\right)\;\sigma_{3}$$

The stress servo-control method is adopted to keep the minor principal stress and the intermediate principal stress ratio constant. To explore the effect of b, six drained true triaxial tests with different b (b=0.0, 0.2, 0.4, 0.6, 0.8 and 1.0) are conducted on particle assemblies. The confining pressure levels simulated in this work are 100 and 200 kPa.

### 2.3. Calibration Procedure

The mechanical properties of DEM samples are controlled by the micro-parameters assigned to the sand particles and the rubber particles. To get the micro-parameters, a calibration procedure is given in this study. Since the rolling resistance model is applied to simulating the behavior of contacting particles, the micro-parameters calibrated in this study are the normal contact stiffness, the shear contact stiffness, friction coefficient, and the rolling friction coefficient. The micro-parameters of the numerical sand–rubber mixtures model, while involving complex interactions between two different materials, i.e., sand to sand, rubber to rubber or sand to rubber, are calibrated by using a strategy as follows. The microscopic parameters of sand–sand contact in pure sand samples are first calibrated by iteratively changing microscopic parameters until the numerical results match the stress–strain curves measured in the laboratory. Then the micro-parameters of sand–rubber contacts are calibrated in samples with rubber content of 10%. Note that this is possible only because the number of rubber–rubber contacts is very small in samples with rubber content of 10%, and rubber–rubber contacts have little influence on the mechanical behavior of samples with 10% rubber particles. The micro-parameters of rubber–rubber contacts are set as the same as those of sand–rubber contacts in this step. The micro-parameters of rubber–rubber contacts are finally calibrated in samples with rubber content of 30% based on the calibrated micro-parameters of sand–sand contacts and sand–rubber contacts. After that, the microscopic parameters are adjusted to be more acceptable. The micro-parameters calibrated in this study are list in Table 2. The density of rubber particle is 1330 kg/m^3^, and the density of sand particles is 2620 kg/m^3^.

**Figure 3:**
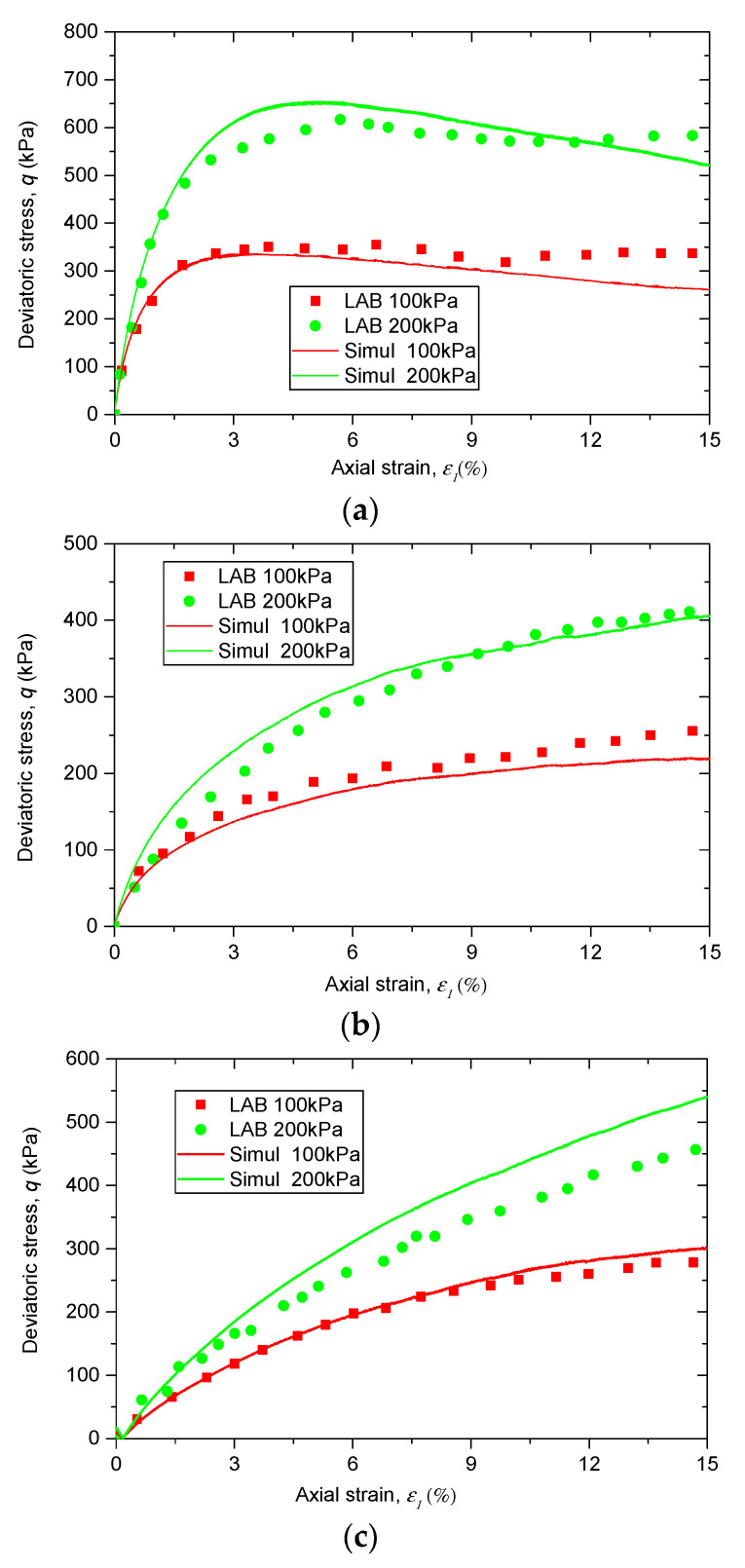
Calibration of simulated results to lab data: stress–strain behaviors of (**a**) pure sand, (**b**) sand–rubber mixtures with 10% rubber particles, and (**c**) sand–rubber mixtures with 30% rubber particles.

Figure 3 presents the comparisons between the experimental data and the corresponding DEM simulation results. It is observed that the stress–strain behavior of the simulation results matches well with the experimental data, suggesting that the calibrated micro-parameters in Table 2 are suitable.

## 3. Results

### 3.1. Macroscopic Behavior

#### 3.1.1. Deviatoric Stress and Volumetric Strain Against the Axial Strain

**Figure 4:**
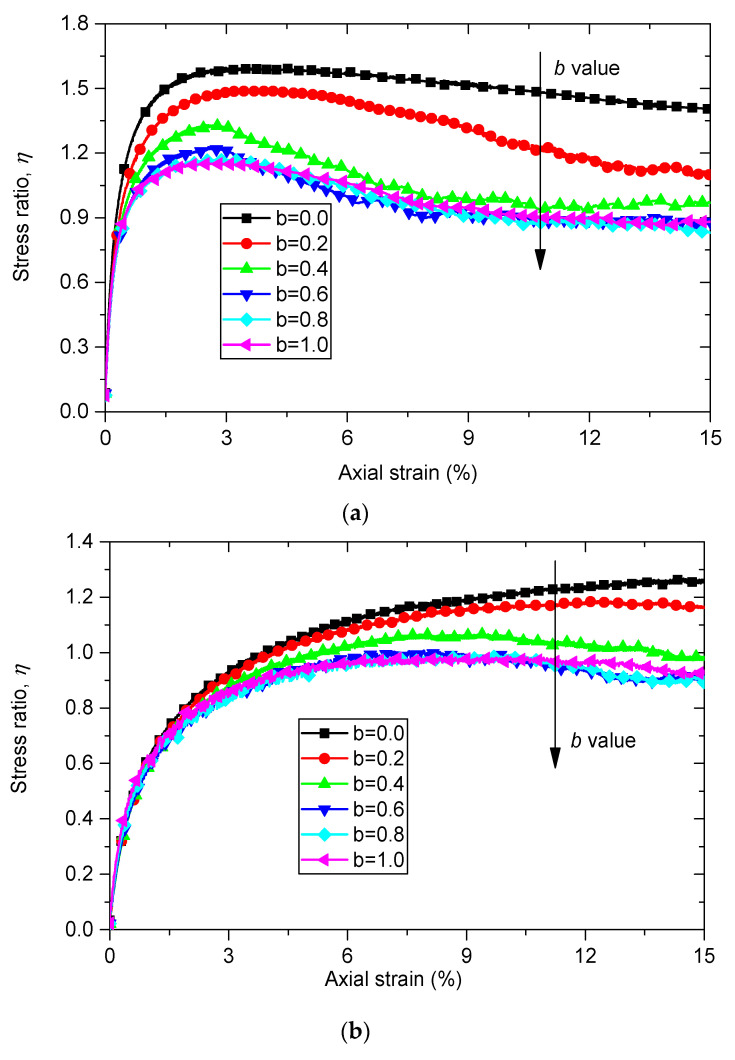

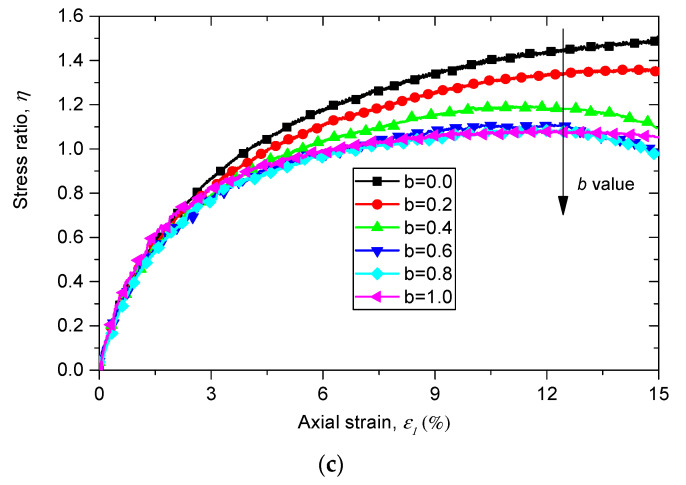
Evolutions of stress ratio η of (**a**) pure sand, (**b**) sand–rubber mixtures with rubber content of 10% and (**c**) sand–rubber mixtures with rubber content of 30% versus major principle strain $\varepsilon_{1}$ with $\sigma_{3}=100\;\mathrm{kPa}$ with different *b* values (*b* is the intermediate principal stress ratio, its definition can be referred to Equation (6).

A series of DEM simulations of true triaxial tests with different rubber contents (0%, 10% and 30%) are conducted with intermediate principal stress ratio b varying from 0.0 to 1.0. The curves of the macroscopic response of the numerical samples under a constant minimum principal stress $\sigma_{3}$ of 100 kPa are shown in Figure 4. The variation trend of b in each curve is marked with an arrow line.

The evolution of stress ratio η=q/p along the axial strain $\varepsilon_{1}$ of pure sand samples with different *b* values is shown in Figure 4a, where  q and p are the deviatoric stress and the mean stress, and can be calculated as:

(7) $$q=\sqrt{{\left(\sigma_{1}-\sigma_{2}\right)}^{2}+{\left(\sigma_{2}-\sigma_{3}\right)}^{2}+{\left(\sigma_{3}-\sigma_{1}\right)}^{2})/2}$$

(8) $$p=\left(\sigma_{1}+\sigma_{2}+\sigma_{3}\right)/3$$

Data in Figure 4a show that the stress ratio q/p is initially independent from the intermediate principal stress ratio *b* until the axial strain $\varepsilon_{1}$ reaches a specific value. After that point the stress ratio η shows a clear dependency on intermediate principal stress ratio *b*. An increase in *b* value leads to a decrease in the value of stress ratio η at the same axial strain $\varepsilon_{1}$. The same trend is also observed in Figure 4b,c, which indicate the evolution of η versus axial strain $\varepsilon_{1}$ for sand–rubber samples with rubber contents of 10% and of 30%, respectively. This observation is also in line with previous experimental and numerical results [20,22,26,28]. By comparing the curves of sand and sand–rubber mixtures with different rubber contents, it can be observed that the pure sand samples show the typical response of strain softening, while sand rubber mixture materials show the response of strain hardening.

#### 3.1.2. Effect of Rubber Content and Intermediate Principal Stress Ratio on the Stress Ratio in the Peak State

**Figure 5:**
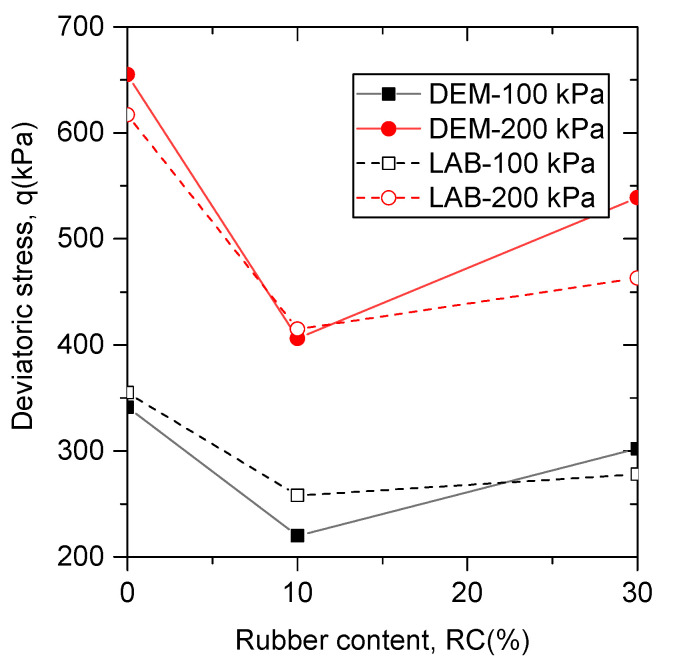
The relationship between peak friction angle and rubber contents for experimental results and DEM simulation results under triaxial compression tests.

Figure 5 shows the comparison between the peak deviatoric stress in experimental tests and that in DEM simulations on specimens (b=0) with various rubber contents under confining pressures of 100 kPa and of 200 kPa. It is observed that the experimental results and the DEM simulation results of the relationship between the peak friction angle and rubber contents share the same trend. The peak friction angle first decreases with the rubber content varying from 0% to 10%, then increases with the rubber content varying from 10% to 30%. This observation can also be found in other experimental results of sand–rubber mixtures containing large rubber particles [49].

**Figure 6:**
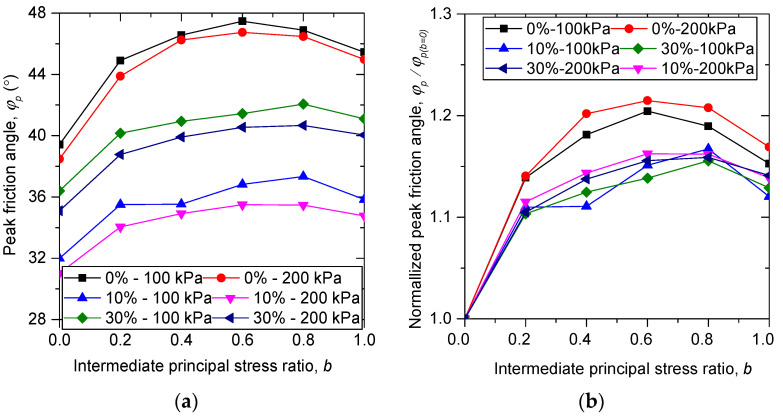
Influence of rubber content on (**a**) the peak friction angle and (**b**) the normalized peak friction angle in true triaxial compression.

The influences of rubber content on the relationship between peak friction angle $\phi_{p}$ and intermediate principal stress ratio *b* are also explored and demonstrated in Figure 6a. The peak friction angle $\phi_{p}$ is the maximum value of the mobilized friction angle $\phi_{m}$ during shearing. The mobilized friction angle can be calculated by:

(9) $$\phi_{m}=sin^{-1}\left((\sigma_{1}-\sigma_{3}\right)/\left(\sigma_{1}+\sigma_{3}\right))$$

As shown in Figure 6a, the peak friction angle of each specimen first increases with intermediate principal stress ratio *b* until a special value is reached, and then decreases with *b*. The samples of b=1.0 have a greater value of peak friction angle than those of b=0.0. This trend is in line with the previous experimental and numerical results available, suggesting that the numerical true triaxial shearing test conducted on sand–rubber mixtures in this study is reasonable. It is noted that the special value of *b* for pure sand is b=0.6, while for sand–rubber mixtures the special value of *b* is b=0.8, and the peak friction angles for sand–rubber samples with b = 0.2, 0.4, 0.6, and 0.8 nearly fall on a straight line, which are quite different from those for pure sand samples. This means that the failure pattern can be changed by the addition of rubber particles, as confirmed in Figure 6b where the responses of sand–rubber mixtures are quite different from those of pure sand when normalized by $\phi_{p}$ for b=0.0.

### 3.2. Micromechanical Response

#### 3.2.1. Coordination Number

**Figure 7:**
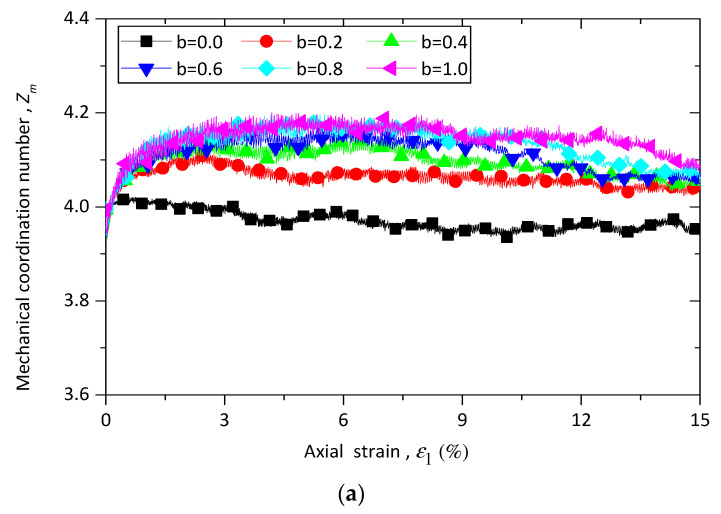

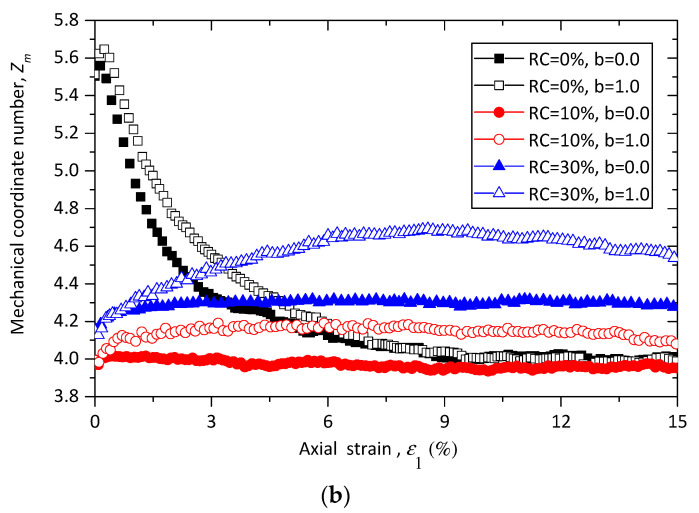
Evolution of the mechanical coordinate number $Z_{m}$ for (**a**) different intermediate stress ratio *b* for sand–rubber mixtures with 10% rubber particles; (**b**) different rubber content under confining stress of 100 kPa.

An important parameter widely used to evaluate the internal contact response is the coordinate number. The coordinate number is the average number of the contacts of each particle. Various authors have noted that in granular materials there are particles with no contact or only one contact and they do not contribute to the force transmission. Thornton [41] defines the mechanical coordination number to neglect these particles as:

(10) $$Z_{m}=\left(2N_{c}-N_{1}\right)/(N_{b}-N_{1}-N_{0})$$

where $N_{c}$ is the number of total contacts, $N_{b}$ is the number of total particles, while $N_{1}$ and $N_{0}$ are the number of particles with one and with zero contacts, respectively. The evolution of the mechanical coordinate number $Z_{m}$ against axial strain $\varepsilon_{1}$ for sand–rubber specimens with different *b* values under constant confining pressure 100 kPa is plotted in Figure 7a. As shown in Figure 7a, all the simulations have an initial value of $Z_{m}=4.0$. $Z_{m}$ increases at the beginning of shearing progresses and then slightly decreases until the termination of shearing. It is also found that an increase in the b value leads to an increase in the value of $Z_{m}$ at the same axial strain $\varepsilon_{1}$. $Z_{m}$ for samples with b=0.0  and b=1.0  are the lower and upper bounds, respectively.

Figure 7b illustrates the evolution of $Z_{m}$ for specimens with different rubber contents under triaxial compression and extension tests. The pure sand samples demonstrate a typical behavior of a dense material, while the sand–rubber samples show a behavior of a loose one. This trend is in agreement with the results shown in Figure 4. It is also observed that $Z_{m}$ of samples with 30% rubber particles is higher than $Z_{m}$ of those with 10% rubber particles. This may be attributed to the fact that with the increase of rubber content, the total number of particles decreases, while the contact number of each particle increases.

**Figure 8:**
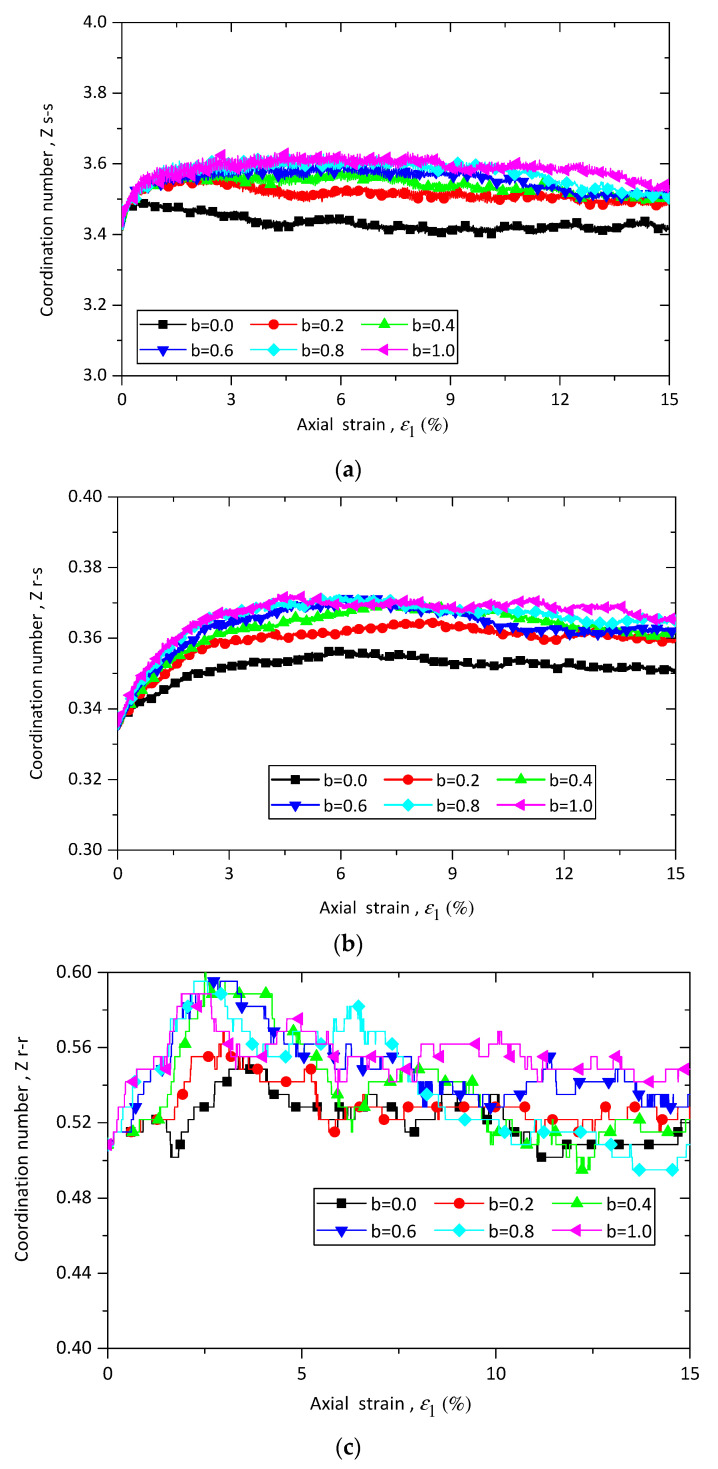
Evolution of coordinate number of (**a**) $Z_{s-s}$, (**b**) $Z_{r-s}$, and (**c**) $Z_{r-r}$ for sand–rubber mixtures with 10% rubber particles under different loading paths.

For binary mixture materials with two types of particles, Minh and Cheng [50] has defined three types of coordinate number relating to each type of contact. Following Minh and Cheng [50], the coordinate numbers of rubber–sand mixture for sand–sand contacts $Z_{s-s}$, rubber–sand contacts $Z_{r-s}$ and rubber–rubber contacts $Z_{r-r}$ are defined as:

(11) $$Z_{s-s}=2N_{c,s-s}/N_{b,s}$$

(12) $$Z_{r-s}=2N_{c,r-s}/N_{b}$$

(13) $$Z_{r-r}=2N_{c,r-r}/N_{b,r}$$

where $N_{c,s-s}$, $N_{c,r-s}$, and $N_{c,r-r}$ are respectively the number of sand–sand, rubber–sand, and rubber–rubber contacts, while $N_{b,s}$ and $N_{b,r}$ are the number of sand and rubber particles. Figure 8 shows the evolution of $Z_{s-s}$, $Z_{r-s}$, and $Z_{r-r}$ for samples with rubber content of 10% in different loading paths with constant confining pressure of 100 kPa. Each contact type of coordinate number has the same initial value. The initial value of $Z_{s-s}$ is the largest, followed by $Z_{r-r}$, and then $\;Z_{r-s}$. $\;Z_{s-s}$ and $\;Z_{r-s}$ are initially independent from intermediate principal stress ratio *b* and quickly divided from each other at a very small axial strain. After that $\;Z_{s-s}$ and $\;Z_{r-s}$ generally increase as b increases at the same axial strain. The values of $Z_{r-r}$ are very volatile because of the limited number of rubber particles and rubber–rubber contacts. It is obvious that because of the limitation of the number of rubber particles, the micro structure and the mechanical behavior are dominated by sand–sand particles when rubber content is 10%.

**Figure 9:**
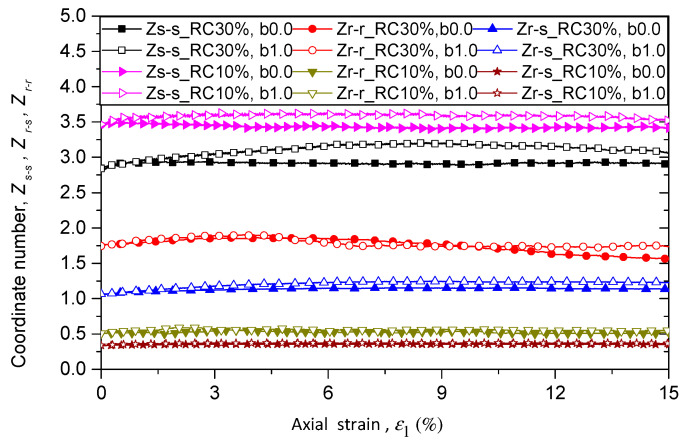
Evolution of coordinate number of $Z_{s-s}$, $Z_{r-s}$, and $Z_{r-r}$ for samples with rubber contents of 10% and 30% under different *b* values.

Figure 9 shows the evolutions of $Z_{s-s}$, $Z_{r-s}$, and $Z_{r-r}$ for samples with rubber content of 10% and 30% under triaxial compression and tension tests. As shown in Figure 9, $Z_{s-s}$ decreases when rubber content increases from 10% to 30%, while $Z_{r-s}$ and $Z_{r-r}\;$ increase with rubber content. The values of $Z_{s-s}\;\mathrm{and}\;Z_{r-s}$ increase with *b* for samples with rubber content of 30%, which agrees with the trend in samples with rubber content of 10%.

#### 3.2.2. Proportion of Strong Contact in Different Types of Contacts

Gong et al. [31] has investigated the role of different contact type in a strong force network and has concluded that rubber–rubber contacts share a very low proportion in the strong network, and contribute less to stress transmission within the system. In this study, we investigate the role of different contact types in a different way. The strong contact ratio is proposed and defined as the ratio of the number of strong contacts to the number of all contacts (sand–sand, sand–rubber, rubber–rubber, and overall contacts). According to Radjai et al. [51] as well as Shi and Guo [52], the contacts can be divided into strong contacts and weak contacts by the average normal contact force, which is the average value of the normal contact force of all contacts.

**Figure 10:**
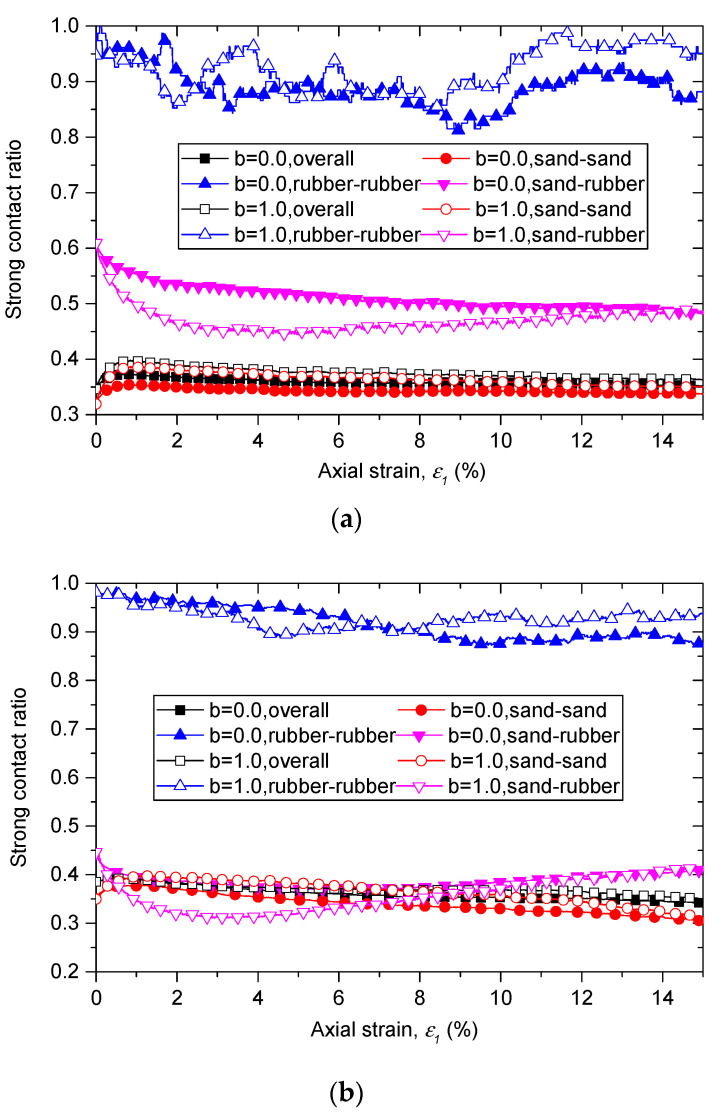
Proportions of strong contact of different type of contacts for sand–rubber mixtures with (**a**) 10% rubber particles; (**b**) 30% rubber particles.

The evolutions of strong contact ratios in different types of contacts with rubber content of 10% and of 30% are shown in Figure 10. For sand–sand contact, the strong contact ratios nearly equal to those of overall contacts at the same axial strain for all samples. The strong contact ratios of rubber–rubber contact present a different performance. They are much higher than those of overall contacts, and their initial values are nearly 1.0, which means nearly all rubber–rubber contacts are strong contacts. When the shearing begins, the strong contact ratios of rubber–rubber contacts present fluctuate declining with axial strain $\varepsilon_{1}$, but their values keep no less than 0.8 for all the samples. This means rubber–rubber contacts mainly participate in strong force networks, and even with rubber content of 10%, the number of rubber particles is very small, rubber particles can significantly affect the micro structure and force transmission in the contact network. This finding also can be used to explain why the failure behavior is changed by adding rubber particles. A suggested explanation of this phenomenon is that the added large rubber particles mainly affect the inherent stability of the strong network.

For the case of sand–rubber contacts, the strong contact ratios are greater than those of overall contacts for samples with 10% rubber particles, while equal to those of overall contacts for samples with 30% rubber particles. This implies that force chains consisting only of rubber particles may exist in the sand–rubber specimens when rubber content exceeds 30%. It should be noted that the coordinate number of rubber–sand contacts increase with b as shown in Figure 8b and Figure 9 in the previous section. Considering the effect of intermediate principal stress ratio on the strong contact ratios and coordinate numbers of rubber–sand contacts together, we can find that with an increase of b, the force transmitted through the rubber particles increases, and more rubber–sand contacts are needed to laterally support the force chain.

#### 3.2.3. Fabric Tensor and Anisotropy

Stress-induced anisotropy is one of the most important properties associated with the key features of granular materials. The concept of fabric, which was first proposed by Oda [53], is a useful statistical method to describe the geometrical anisotropy induced by applied stress in granular materials. The fabric anisotropy can be quantified by a fabric tensor based on the contact normal distribution. The fabric tensor of the whole contact networks is defined by Satake [54] as:

(14) $$\phi_{ij}\;=\;\frac{1}{N_{c}}\;\overset{\;N_{c}}{\underset{k\;=\;1}{\sum }}\;n_{i}^{k}\;n_{j}^{k}$$

where $n_{i}^{k}$ is the unit contact normal component in the ith direction.

**Figure 11:**
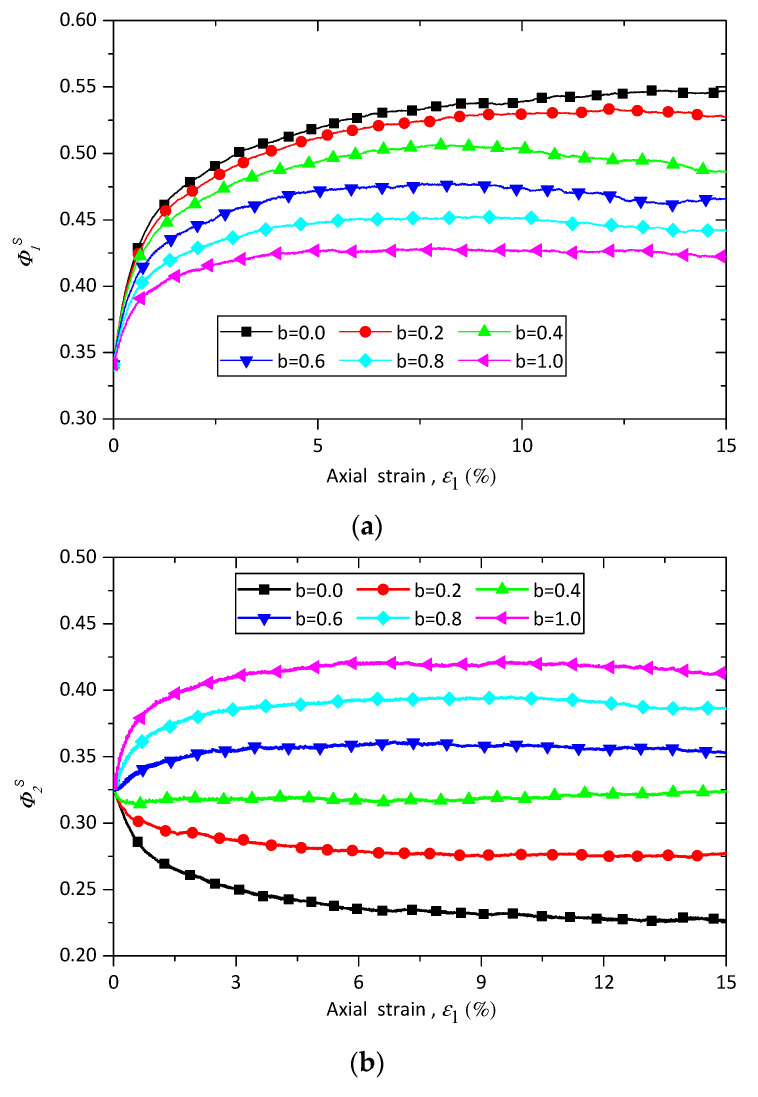

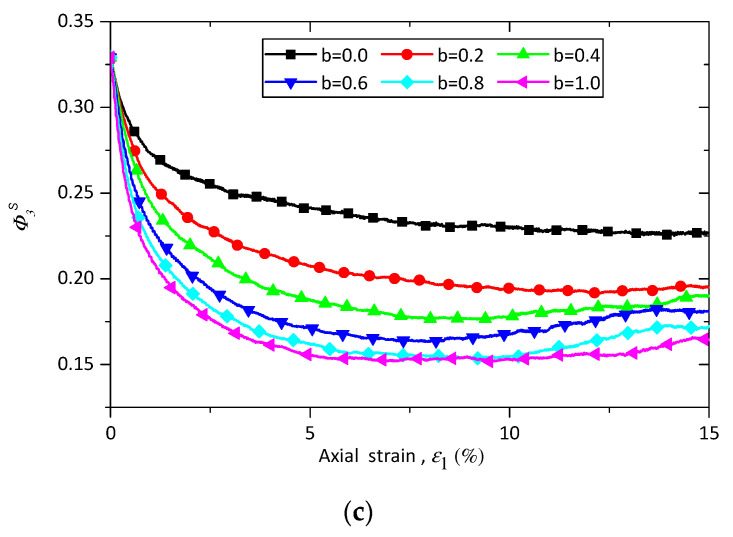
Evolution of (**a**) $\phi_{1}^{s}$, (**b**) $\phi_{2}^{s}$, and (**c**) $\phi_{3}^{s}$ for the samples of 10% rubber particles with various b under confining pressure of 100 kPa.

Similar to the fabric tensor, the fabric tensor of strong contacts and weak contacts can be defined as:

(15) $$\phi_{ij}^{s}\;=\;\frac{1}{N_{c}^{s}}\;\overset{N_{c}^{s}}{\underset{k\;=\;1}{\sum }}n_{i}^{k}\;n_{j}^{k}$$

(16) $$\phi_{ij}^{w}\;=\;\frac{1}{N_{c}^{w}}\;\overset{N_{c}^{w}}{\underset{k\;=\;1}{\sum }}\;n_{i}^{k\;}n_{j}^{k}\;$$

where $N_{C}^{s}$ and $N_{C}^{w}$ are respectively the numbers of strong and weak contacts. $\phi_{1}^{s}$, $\phi_{2}^{s}$, and $\phi_{3}^{s}$ are the principal values of the fabric tensor of strong contacts associated with the directions of maximum, intermediate, and minimum principal stresses, respectively. Figure 11 shows the evolutions of $\phi_{1}^{s}$, $\phi_{2}^{s}$, and $\phi_{3}^{s}$ of sand–rubber samples with rubber content of 10% under constant confining stress of 100 kPa. $\phi_{1}^{s}$ first increases with axial strain and then nearly keeps constant, and an increase in the value of *b* leads to a decrease in the $\phi_{1}^{s}$. The value of $\phi_{2}^{s}$ first decreases with the axial strain until *b* reaches 0.4, and after that the value of $\phi_{2}^{s}$ increases with the axial strain (Figure 11b). It is also seen that the value of $\phi_{2}^{s}$ rises as the intermediate principal stress ratio *b* increases. The evolution of $\phi_{3}^{s}$ has the reverse trend compared with that of $\phi_{1}^{s}$: its value first decreases with the axial strain and then remains nearly constant. On the contrary, the intermediate principal stress ratio *b* has the same effect on the evolutions of $\phi_{3}^{s}$ and $\phi_{1}^{s}$, and an increase in the value of *b* leads to a decrease in the $\phi_{3}^{s}$.

**Figure 12:**
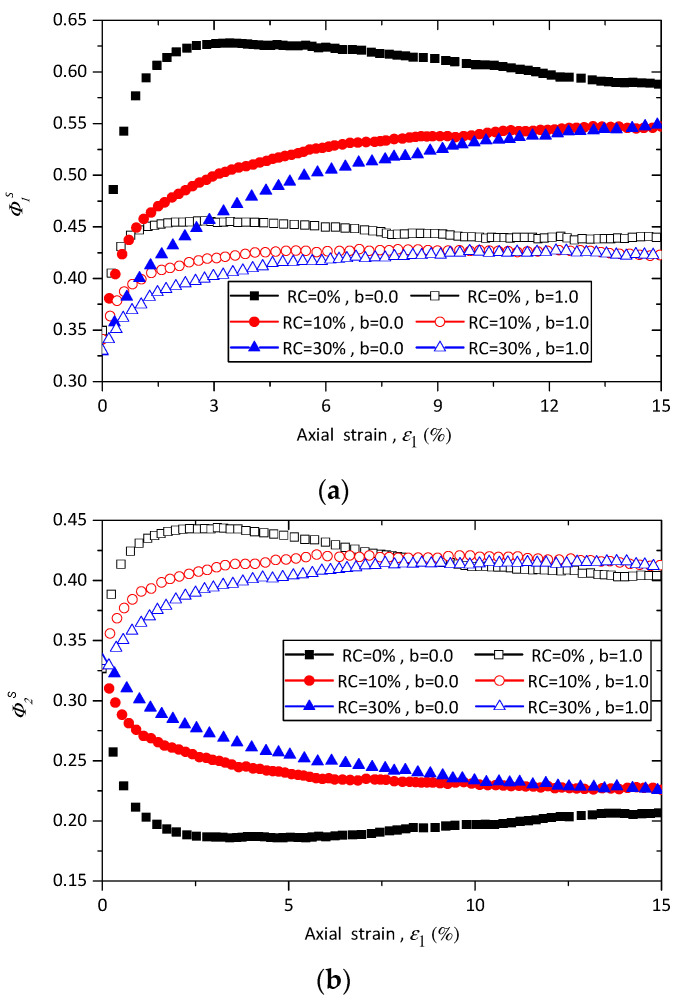

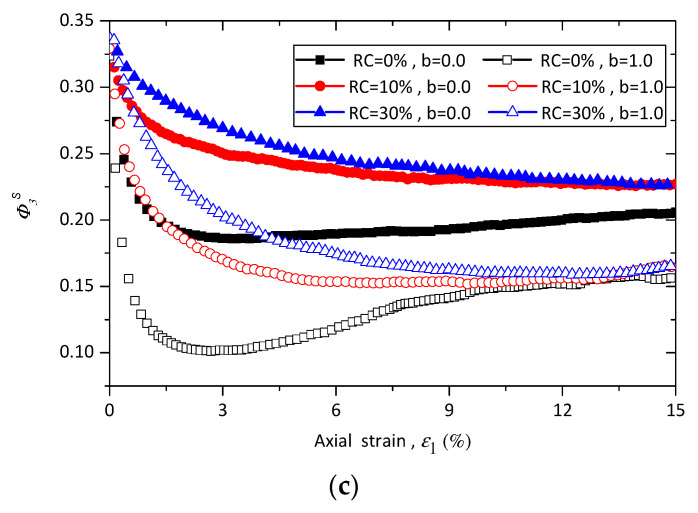
Evolution of (**a**) $\phi_{1}^{s}$, (**b**) $\phi_{2}^{s}$, and (**c**) $\phi_{3}^{s}$ for samples with different rubber content under triaxial compression and tension conditions.

To clarify the effect of rubber content on the principal values of fabric tensor of strong contacts, Figure 12 presents the evolutions of $\phi_{1}^{s}$, $\phi_{2}^{s}$, and $\phi_{3}^{s}$ for samples with 0%, 10%, and 30% rubber particles under triaxial compression and tension conditions. As shown in Figure 12a, the value of $\phi_{1}^{s}$ of pure sand increases with the axial strain until a peak value is reached, and then slightly decreases to the end of shearing, while the behavior of sand–rubber mixtures has the same trend mentioned before. It is noted that the value of $\phi_{1}^{s}$ is first dependent on rubber content until the axial strain reaches a certain value, and after that the $\phi_{1}^{s}$ is nearly independent from the rubber content. Similar trends are observed in the evolutions of $\phi_{2}^{s}$ and $\phi_{3}^{s}$, as shown in Figure 12b,c.

**Figure 13:**
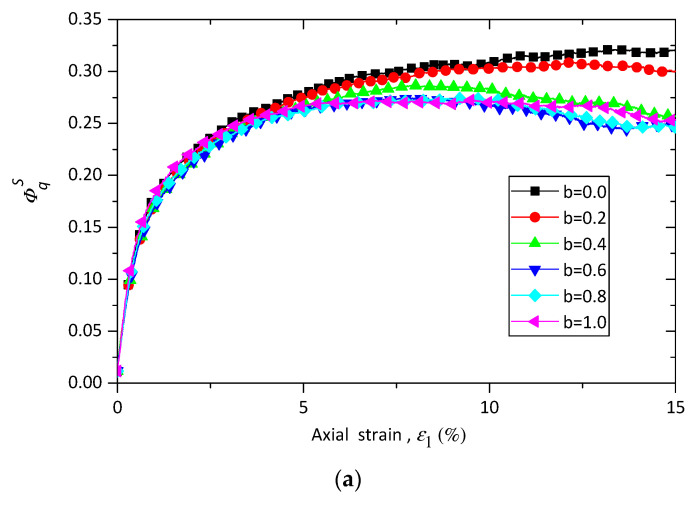

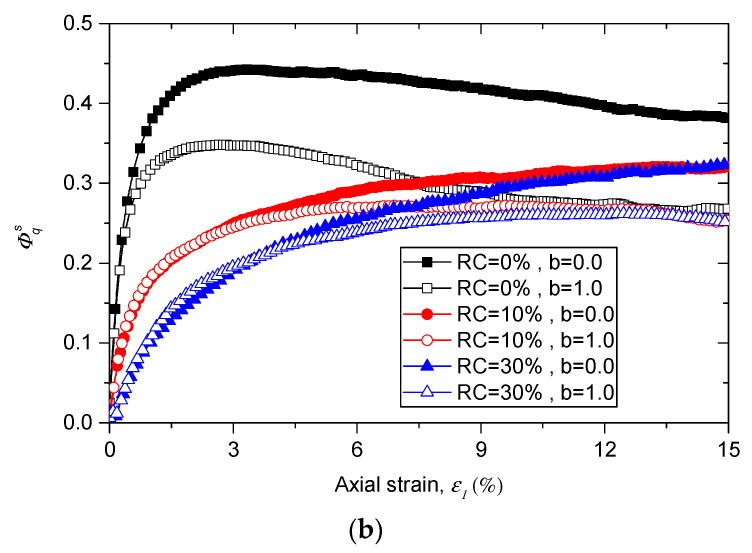
Effect of rubber content on the evolution of deviatoric fabric of strong contact for (**a**) samples with 10% rubber particles under various *b* values; and (**b**) for samples with different rubber contents under triaxial compression and triaxial tension tests.

The evolution of the strong deviatoric fabric for the samples with 10% rubber particles under different stress conditions are presented in Figure 13a. The deviatoric fabric is initially independent on the intermediate principal stress ratio *b* until the axial strain reaches nearly 5%. After that the deviatoric fabric becomes dependent on the *b* values, and an increase in *b* value leads to a decrease in the value of deviatoric fabric. The effects of rubber content on the evolutions of the deviatoric fabric of strong contacts is shown in Figure 13b. It can be found that a higher rubber content leads to a lower value of deviatoric fabric. This means the samples with a higher rubber content shows lower level of fabric anisotropy.

**Figure 14:**
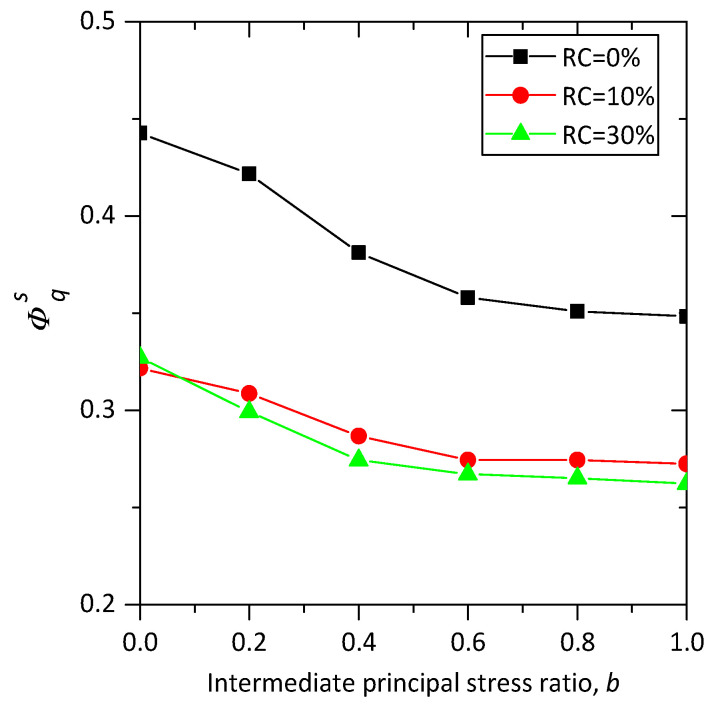
Effect of rubber content on the relationship between maximum deviatoric fabric and intermediate principal stress ratio.

Figure 14 shows the relationship between the peak deviatoric fabric and the intermediate principal stress ratio *b* of samples with different rubber content under confining pressure of 100 kPa. It can be observed that the peak deviatoric fabric decreases with intermediate principal stress ratio *b* for all the samples, and the peak deviatoric fabric of pure sand is higher than those ones of sand–rubber samples with the same *b* values. The samples with rubber content of 10% and of 30% nearly have the same peak value of deviatoric fabric.

#### 3.2.4. Normal Contact Force and the Probability Density Function of Normal Contact Force

The probability density function (PDF) of contact force can be used as a statistical tool to explore the characteristic of the force transmission behavior in granular materials. The contact forces are partitioned into strong contact forces and weak contact forces by the average contact force. The probability of the strong forces is presented as an exponential equation, while the probability of the weak forces is described by a power equation, as follows:

(17) $$P\left(f_{x}\right)=\{\begin{array}{c} e^{-A\left(\frac{\left|f_{x}\right|}{\langle |f_{x}|\rangle }\right)},\;\;\;\;\;f_{x}>\;\langle |f_{x}|\rangle \\ {\left(\frac{\left|f_{x}\right|}{\langle |f_{x}|\rangle }\right)}^{B},\;\;f_{x}\leq \;\langle |f_{x}|\rangle \end{array}$$

where $f_{x}$ is the normal contact force or shear contact force; $\langle |f_{x}|\rangle$ is the average value of $\left|f_{x}\right|$; *A* and *B* are the parameters that imply the inhomogeneity of forces in discrete element model.

**Figure 15:**
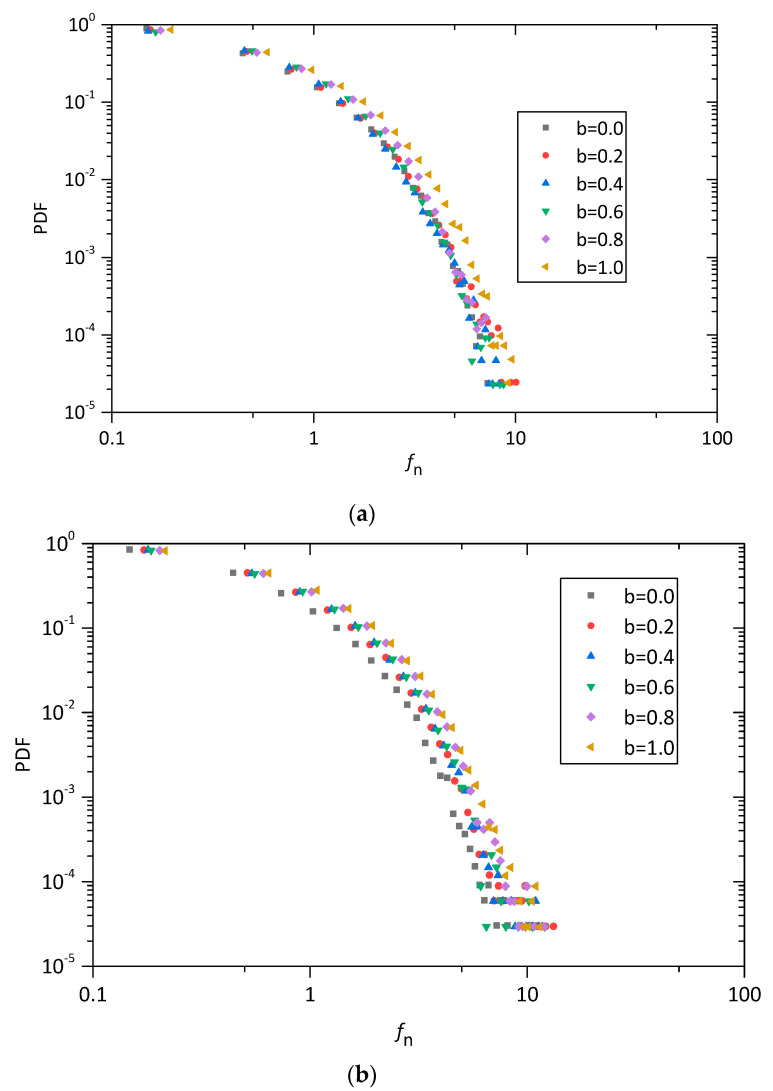

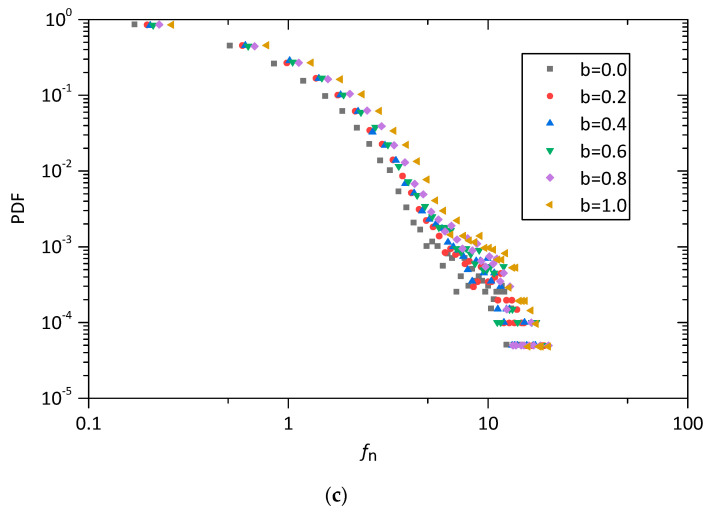
Probability density function of $f_{n}$: (**a**) pure sands, (**b**) sand–rubber mixtures with 10% rubber particles, and (**c**) sand–rubber mixtures with 30% rubber particles.

Figure 15 shows the probability density function (PDF) of the normal contact forces at the shear strain of 15% for pure sand and sand–rubber mixtures with different intermediate principal stress ratio in a log-linear space. It is clear that the normal contact forces show a high agreement with the probability density distribution. It can also been found, the probability density decreases with the contact forces, and the magnitude of the contact forces increases with *b* values.

**Figure 16:**
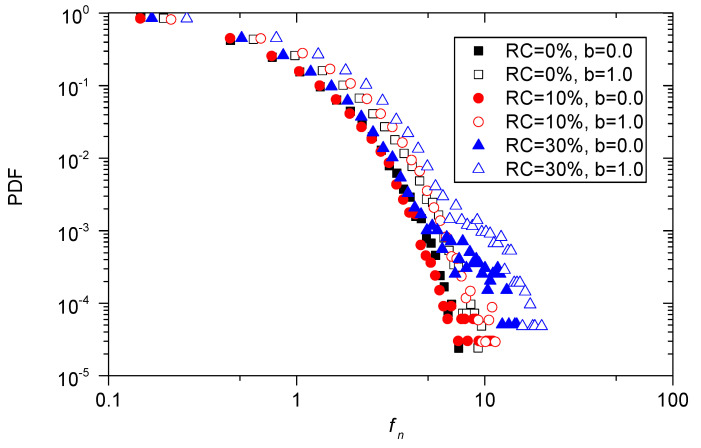
Probability density function of $f_{n}$ for pure sand and sand–rubber mixtures with 10% and 30% rubber particles under triaxial compression and triaxial tension tests.

The probability density distributions of pure sand and sand–rubber mixtures under the true triaxial loading paths of b=0 and b=1.0 are plotted in Figure 16. As shown in Figure 16, the rubber content has a significant effect on the probability density distributions of sand–rubber mixtures. A wider distribution of contact force in the sample is shown as the rubber content increases, and the discrepancies in the probability density distribution of normal contact force associated with *b* = 1.0 are larger than the ones associated with b=0.0.

**Figure 17:**
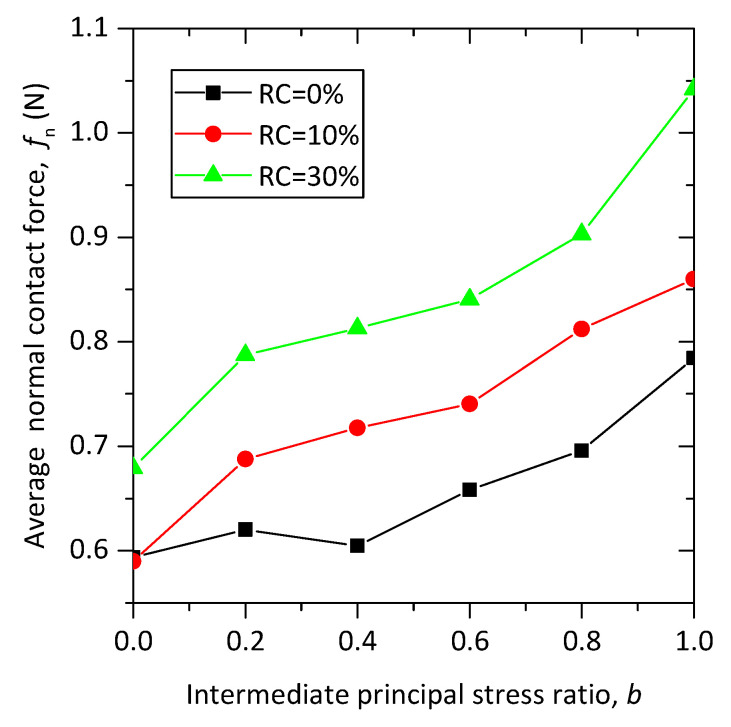
The relationship between average normal contact force and *b* for pure sand and sand–rubber mixtures.

The average normal contact force is defined as the average value of the normal contact force of all contacts. The average normal contact forces of samples with different rubber contents under various loading paths at the axial strain of 15% are shown in Figure 17. It can be seen that for the samples with the same rubber content, the average normal contact forces increase with *b* values, and for the same *b* value, the average normal contact forces increase with rubber content.

## 4. Conclusions

Several DEM simulated true triaxial shear tests have been conducted to investigate the effect of intermediate principal stress ratio on the mechanical behavior of sand–rubber mixtures. Both the macroscale and microscale performance has been discussed. The main conclusions drawn from the numerical study are the following:

1. The peak strengths of samples under conventional triaxial tests first decrease with 10% rubber particles added, and then increase when the proportion of rubber particles rises up to 30%, but the peak strengths sand–rubber mixtures with either 10% or 30% rubber particles are lower than those of pure sand. This trend is in agreement with previous experimental results and numerical simulations on sand–rubber mixtures with large rubber particles, which confirms the feasibility of the simulations conducted in this study. The same trend can also be observed in the peak friction angles for the samples at each intermediate principal stress ratio.
2. For sand–rubber mixtures, the relationship between the peak friction angle and the intermediate principal stress ratio is quite different from that for pure sand, which means adding rubber particles can change the failure behavior of sand under complex loading conditions. A suggested explanation of this phenomenon is that the added large rubber particles mainly affect the inherent stability of the strong network. This study can provide a reference for the constitutive model development of sand–rubber mixtures.
3. The investigation on the strong contact ratio of different types of contacts show that nearly all of the rubber–rubber contacts of sand–rubber mixtures are strong contacts, no matter what the rubber contents and the values of intermediate principal stress ratio are. While the strong contact ratio of rubber–sand contacts is higher than that of overall contacts for specimens with 10% of rubber particles, and becomes nearly equal to that of the overall contacts when rubber content rises up to 30%. It can be concluded that rubber content can significantly influence the micro structure and the force transmission in the contact network. This finding also confirms the explanation in the Conclusion 2.
4. For samples with the same rubber content, the strong contact ratio of rubber–sand contacts decrease with the principal stress ratio *b*, while the coordinate number of rubber–sand contacts increase with *b*. It means that with an increase of *b*, the force transmitted through the rubber particles increases, and more rubber–sand contacts are needed to support the force chain.
5. The analysis of the fabric anisotropy shows that the deviatoric fabric of strong contacts demonstrates a decline by adding large rubber particles, and the deviatoric fabric of strong contacts also decreases with *b*, which is in line with the previous numerical simulations.

## Figures and Tables

**Table 1:** List of simulations performed in this study.

Set Test ID	b Values	Rubber Content (%)	Sand Particles	Rubber Particles
CS-100-b	0.0, 0.2, 0.4, 0.6, 0.8, 1.0	0	43,693	0
CS-200-b	0.0, 0.2, 0.4, 0.6, 0.8, 1.0	0	43,693	0
RS-10-100-b	0.0, 0.2, 0.4, 0.6, 0.8, 1.0	10	34,909	299
RS-10-200-b	0.0, 0.2, 0.4, 0.6, 0.8, 1.0	10	34,909	299
RS-30-100-b	0.0, 0.2, 0.4, 0.6, 0.8, 1.0	30	18,995	631
RS-30-200-b	0.0, 0.2, 0.4, 0.6, 0.8, 1.0	30	18,995	631

**Table 2:** Numerical micro-parameters used in this study.

Parameters	Values			
	Rubber–Rubber Contact	Sand–Sand Contact	Rubber–Sand Contact	Wall-Particle Contact
Effective modules	3.5 × 10^4^	1.0 × 10^8^	8.0 × 10^6^	1.0 × 10^8^
Normal to shear stiffness ratio	1.0	1.0	1.0	1.0
Inter-particle friction coefficient	1.5	0.335	0.5	0.0
Rolling friction coefficient	1.0	0.35	0.5	N/A
Damping coefficient	0.7	0.7	0.7	N/A

## Nomenclature

RSM	Sand–rubber mixtures
DEM	Discrete element method
PSD	Particle size distribution
RC	Rubber content
PDF	Probability density function
$k^{n}$	The normal stiffness
$k_{s}$	The shear stiffness
μ	The friction coefficient
$M^{r}$	The rolling resistance moment
$k_{r}$	The rolling resistance stiffness
$\mu^{r}$	The rolling friction coefficient
$\sigma_{1}$	The major principal stress
$\sigma_{2}$	The intermediate principal stress
$\sigma_{3}$	The minor principal stress
$\varepsilon_{1}$	The major principle strain
q	The deviatoric stress
p	The mean stress
b	The Intermediate principal stress ratio
$\phi_{p}$	The peak friction angle
$\phi_{m}$	The mobilized friction angle
$Z_{m}$	The mechanical coordination number
$Z_{s-s}$	The coordination number of sand–sand contacts
$Z_{r-s}$	The coordination number of rubber–sand contacts
$Z_{r-r}$	The coordination numbers of rubber–rubber contacts
$\phi_{1}^{s}$	Major principal fabric of strong contacts
$\phi_{2}^{s}$	Intermediate principal fabric of strong contacts
$\phi_{3}^{s}$	Minor principal fabric of strong contacts
$\phi_{q}^{s}$	Strong deviatoric fabric
